# Awareness, Utilization, Satisfaction, and Out-of-Pocket Expenditure of Ayushman Bharat Pradhan Mantri Jan Arogya Yojana in Rural Community: A Cross-Sectional Study in Meerut

**DOI:** 10.7759/cureus.110091

**Published:** 2026-06-02

**Authors:** Anshul Kumar, Pawan Parashar, Rijul Ranjan, Kaynat Nasser, Manvi Vasudev, Yashendra Sethi

**Affiliations:** 1 Community Medicine, Subharti Medical College, Swami Vivekanand Subharti University, Meerut, IND; 2 Medicine and Surgery, Subharti Medical College, Swami Vivekanand Subharti University, Meerut, IND; 3 Internal Medicine, Subharti Medical College, Swami Vivekanand Subharti University, Meerut, IND

**Keywords:** ayushman bharat, ayushman bharat pradhan mantri jan arogya yojana (ab pmjay), health insurance, out-of-pocket expenditure, universal health coverage (uhc)

## Abstract

Background

Ayushman Bharat Pradhan Mantri Jan Arogya Yojana (AB-PMJAY) represents India’s flagship publicly funded health insurance initiative aimed at advancing universal health coverage by improving access to secondary and tertiary care while reducing out-of-pocket expenditure (OOPE). Despite its scale and policy significance, real-world evidence on awareness, utilization, patient experience, and residual financial burden in rural settings remains limited.

Objectives

To assess the awareness, utilization, satisfaction, and OOPE associated with AB-PMJAY among rural beneficiaries in the district of Meerut, Uttar Pradesh.

Methods

A community-based cross-sectional study was conducted from November 2024 to November 2025 across four randomly selected villages within a 10-km rural radius of Subharti Medical College, Meerut. A total of 208 households possessing and utilizing AB-PMJAY cards were included. Data were collected through house-to-house interviews using a pretested semi-structured questionnaire. Key outcome variables included awareness (scheme knowledge, empanelled hospitals, grievance redressal), utilization (enrolment duration, documentation, coverage), satisfaction (clinical care, staff behaviour, administrative services), and OOPE.

Results

Overall awareness of AB-PMJAY was high (96.6%), and most participants were aware of scheme benefits (96.2%) and service availability in both public and private hospitals (94.7%). However, awareness of empanelled hospitals (30.3%) and grievance redressal mechanisms (26.4%) remained low. All participants reported utilizing the scheme, with 43.8% enrolled for more than three years. Most beneficiaries (74.0%) reported coverage utilization up to Rs. 1 lakh. Despite insurance coverage, 100% of participants incurred OOPE, though the majority (78.8%) spent less than Rs. 10,000. Additionally, awareness alone may not be sufficient to prevent OOPE and may reflect a more complex pattern of healthcare use and hidden costs. Satisfaction with clinical services (97.2%) and healthcare staff behaviour (99%) was high; however, satisfaction with administrative aspects such as file management (17.8%) and food quality (14.4%) was substantially lower. Socio-demographic factors, including education, caste, and socioeconomic status, were significantly associated with awareness indicators.

Conclusion

While AB-PMJAY demonstrates high awareness, substantial utilization, and strong satisfaction with clinical care among rural beneficiaries, critical gaps persist in functional awareness, particularly regarding empanelled facilities and grievance mechanisms. Importantly, persistent OOPE across all beneficiaries highlights incomplete financial risk protection. Strengthening beneficiary education, improving administrative efficiency, and addressing hidden healthcare costs are essential to optimize the scheme’s impact.

## Introduction

Health insurance protects individuals from catastrophic medical costs while improving access to affordable healthcare [1]. With growing recognition of financial risk protection as a core health-system function, the global health insurance market, valued at USD 2.5 trillion in 2024, is projected to reach USD 5.12 trillion by 2034 [2].

Within this context, Universal Health Coverage (UHC) has emerged as a central policy priority worldwide. UHC embodies the principle that all individuals should have access to comprehensive health services, including promotive, preventive, curative, rehabilitative, and palliative care of adequate quality without incurring financial hardship [3]. Financial protection is a cornerstone of UHC and represents one of its primary objectives. Inadequate financial protection mechanisms lead to substantial out-of-pocket (OOP) expenditures, which not only impose economic strain but also act as barriers to healthcare access. Effective health systems therefore rely on prepayment and risk pooling mechanisms, rather than point-of-care payments, to safeguard individuals against financial distress [4]. India’s healthcare system represents a complex interplay of public and private sector providers serving a population exceeding 1.4 billion. Despite significant reforms, the system continues to face persistent structural and financial challenges [5]. Insurance coverage remains limited, particularly in rural areas, where only about 14% of the population is covered, compared to 18% in urban settings [6]. Consequently, an estimated 400 million individuals lack any form of financial risk protection, perpetuating cycles of poverty and limiting socioeconomic mobility among vulnerable groups [7]. Healthcare expenditures push approximately 32-39 million individuals below the poverty line annually, reinforcing the bidirectional relationship between illness and impoverishment [8].

In response to these challenges, the Government of India introduced the National Health Policy in 2017, outlining a pathway toward UHC through reduced reliance on OOP expenditure and expansion of publicly financed healthcare coverage [9]. This vision materialized in the form of the flagship National Health Protection Mission, popularly known as Ayushman Bharat Pradhan Mantri Jan Arogya Yojana (AB-PMJAY). The programme comprises two key pillars: Health and Wellness Centres (HWCs), which deliver comprehensive primary care, and the Pradhan Mantri Jan Arogya Yojana (PM-JAY), which provides financial protection for secondary and tertiary healthcare services. Launched on 23 September 2018, PM-JAY is the world’s largest publicly funded health insurance scheme, covering approximately 550 million beneficiaries-nearly 40% of India’s population. It offers an annual cashless benefit of Rs. 5 lakh per household for specified procedures across empanelled public and private hospitals. Key features include nationwide portability, absence of restrictions on family size, age, or pre-existing conditions, and equity-based eligibility determined through the Socio-Economic Caste Census (SECC) 2011 criteria [10].

Emerging evidence suggests that PM-JAY has positively influenced healthcare utilization and reduced the financial burden of illness among beneficiaries. Studies report substantial reductions in OOP expenditure, particularly for inpatient and catastrophic care, with the scheme covering up to 90% of medical costs for included services, thereby mitigating distress financing and medical impoverishment [11]. Furthermore, the programme has been associated with improved treatment-seeking behaviour among economically disadvantaged populations, indicating enhanced access to healthcare services [12]. Despite these promising findings, gaps remain in understanding the scheme’s real-world performance at the community level, particularly in rural settings.

The present study aims to evaluate beneficiary experiences and beneficiary-reported implementation-related challenges under AB-PMJAY in a rural community setting. Specifically, the study seeks to assess functional awareness regarding scheme provisions among beneficiaries who have utilized AB-PMJAY services, evaluate patterns of healthcare utilization and beneficiary satisfaction, and estimate the residual OOPE incurred during utilization of scheme benefits.

## Materials and methods

Study design and setting

This community-based cross-sectional survey was conducted from November 2024 to November 2025 in the rural field practice area of a tertiary care teaching institution in northern India. Villages were selected using simple random sampling (lottery method) to improve representativeness and minimize geographical selection bias. The survey was designed to assess utilization patterns, beneficiary experiences, and perceived benefits among households already enrolled in and utilizing AB-PMJAY services. Therefore, only households possessing a valid AB-PMJAY card and having utilized scheme benefits at least once were included. Consequently, awareness findings reflect beneficiary-level awareness rather than community-level prevalence. To ensure methodological uniformity and minimize interviewer-related and temporal variability, all interviews were conducted by the same investigator using a structured questionnaire during a single visit to each eligible household. Although data collection extended across the study period because multiple households from different villages were surveyed, interview methods and data collection procedures remained consistent throughout. In addition, AB-PMJAY policy provisions, eligibility criteria, and utilization processes remained stable during the study period and were not influenced by any known internal or external policy changes that could substantially affect participant responses or scheme utilization patterns. Ethical approval was obtained from the Institutional Ethics Committee, and the study was conducted in accordance with the Declaration of Helsinki.

Operational definitions

For the present study, the following operational definitions were used:

Awareness Regarding AB-PMJAY

Awareness was defined as beneficiary knowledge regarding key provisions of the AB-PMJAY, including awareness of scheme benefits, empanelled hospitals, availability of services in public and private hospitals, grievance redressal mechanisms, and healthcare coverage under the scheme. Awareness was assessed using structured yes/no questionnaire items.

Beneficiary Satisfaction

Beneficiary satisfaction referred to the participant’s self-reported level of satisfaction regarding healthcare services received under AB-PMJAY, including clinical care, behaviour of healthcare providers, administrative services, file management, and supportive services. Satisfaction was assessed using categorical responses classified as satisfied, neutral, or dissatisfied.

OOPE

OOPE was defined as any expenditure incurred by the beneficiary despite utilization of AB-PMJAY services during the most recent hospitalization or treatment episode recalled by the participant. This included direct medical expenditure (such as medicines, diagnostics, and procedures not covered under the scheme) as well as direct non-medical expenditure (including transportation, food, and accommodation).

Implementation-Related Challenges

Implementation-related challenges referred to beneficiary-reported difficulties or deficiencies experienced during utilization of AB-PMJAY services, including limited operational awareness, difficulties related to healthcare access, persistence of OOPE despite insurance coverage, and dissatisfaction with administrative or supportive services.

Utilization of AB-PMJAY Services

Utilization referred to availing healthcare services under AB-PMJAY by beneficiaries possessing a valid scheme card and having used scheme benefits at least once at an empanelled healthcare facility.

Study population and eligibility criteria

The study population consisted of households residing in the selected rural area with at least one member enrolled under AB-PMJAY. The unit of analysis was an adult individual (aged ≥18 years) who had utilized services under the scheme.

Inclusion Criteria

Participants were eligible if they (i) were permanent residents of the selected study area, (ii) possessed a valid AB-PMJAY card, (iii) had utilized scheme benefits at least once, and (iv) provided informed written consent to participate.

Exclusion Criteria

Households were excluded if no eligible respondent was available after reasonable attempts, if respondents were unable to provide reliable information, or if consent was declined. Locked households or those without any adult member present at the time of visit were also excluded.

Sample size determination and sampling strategy 

The sample size was calculated based on an expected prevalence of awareness regarding AB-PMJAY of 65%, derived from previously published literature. Using the standard formula:

(\displaystyle n = \frac{Z^2 \times p \times q}{d^2})

where Z=1.96 (95% confidence level), p=65, q=35, and allowable relative error (d)=10% of p, the calculated sample size was approximately 206. To account for potential non-response and ensure balanced representation across selected clusters, the final sample size was rounded to 208 households.

A multistage sampling approach was employed. Initially, villages were selected through simple random sampling. Subsequently, a line listing of eligible households was prepared in each selected village, and households were selected using systematic random sampling. Within each household, one eligible respondent was interviewed.

**Figure 1:**
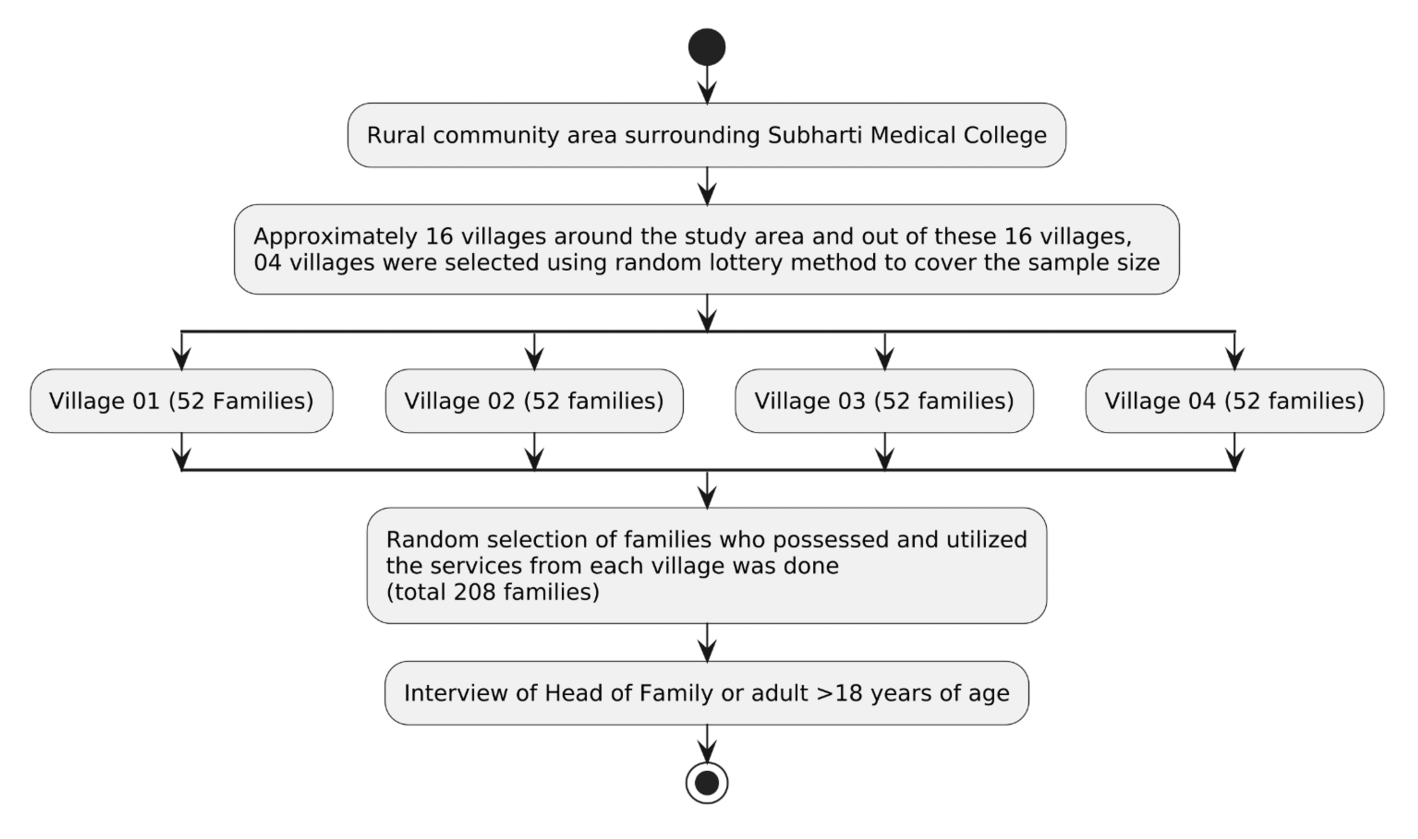
Participant flowchart illustrating the selection of families from four villages in the rural community area of Meerut A total of 208 families who possessed and utilized the services of AB-PMJAY were included in the study. The figure was created using Microsoft Word.

Figure 1 shows a flowchart of the selection of study participants.

Data collection tools and procedures

Data were collected through house-to-house visits using a semi-structured, pretested, and pre-validated questionnaire developed after an extensive review of relevant literature and in accordance with the study objectives. The questionnaire was initially prepared in English, translated into the local vernacular language, and subsequently back-translated to ensure linguistic accuracy, conceptual equivalence, and consistency. Content validity of the instrument was assessed by experts in community medicine and public health. A pilot study was conducted in a population comparable to the study setting to evaluate clarity, comprehensibility, reliability, and contextual applicability of the questionnaire, following which necessary modifications were incorporated.

To ensure uniformity and minimize interviewer-related variability, all interviews and data collection procedures were conducted directly by the primary investigator. During each household visit, every question included in the questionnaire was carefully explained in the local language to the respondents to ensure accurate comprehension and appropriate interpretation. Adequate time was provided for clarification of doubts wherever required, thereby ensuring correct conveyance of information and minimizing the possibility of misunderstanding, response error, and data collection bias. Standardized interviewing techniques were consistently employed throughout the study to improve the reliability and validity of responses. Face-to-face interviews were conducted with eligible respondents during household visits. In each household, the head of the family was interviewed whenever available; in their absence, the eldest or most informed adult member was selected as the respondent.

The questionnaire captured information across multiple domains, including sociodemographic characteristics such as age, sex, religion, marital status, education, occupation, and socioeconomic status, assessed using the modified B.G. Prasad socioeconomic classification. Information regarding awareness of AB-PMJAY included knowledge about scheme benefits, empanelled hospitals, service availability, eligibility criteria, and grievance redressal mechanisms. Utilization-related variables included duration of enrolment, documentation used, types of healthcare services availed, and extent of coverage under the scheme. Data regarding OOPE included both direct medical and non-medical expenditures incurred despite utilization of scheme benefits. Participant satisfaction was assessed across domains, including perceived quality of healthcare services, behaviour of healthcare providers, administrative procedures, and supportive services received during hospitalization. Strict confidentiality of participant information was maintained throughout the study. No personally identifiable information was included in the final analytical dataset. All collected data were stored in password-protected electronic systems accessible only to authorized members of the research team.

Variables and definitions

The primary outcome variables included awareness, utilization, satisfaction, and OOPE related to AB-PMJAY. Awareness was defined as self-reported knowledge of the scheme and its components. Utilization referred to the use of AB-PMJAY services for healthcare needs. Satisfaction was assessed using categorical responses reflecting patient experience across service domains. OOPE was defined as any expenditure incurred by the beneficiary despite scheme coverage.

Independent variables included sociodemographic characteristics such as age, gender, religion, caste, education, occupation, and socioeconomic status.

Statistical analysis

Data were coded, entered, cleaned, and analyzed using SYSTAT Statistical Software version 13.2 (SYSTAT Software Inc., San Jose, California, USA, 2013). Data validation and consistency checks were performed before statistical analysis to ensure accuracy and completeness of the dataset. Descriptive statistics were used to summarize the study variables. Categorical variables were expressed as frequencies and percentages [n (%)], while continuous variables, wherever applicable, were presented as mean ± standard deviation or median with interquartile range depending on data distribution. Inferential statistical analysis was performed to evaluate associations between independent variables and outcome measures. The chi-square (χ²) test was used to compare categorical variables between groups. To control for type I error due to multiple comparisons, the Bonferroni correction was applied, and the likelihood ratio test was used as an alternative measure of association. Multivariable analysis was performed using binary logistic regression. A two-tailed p-value <0.05 was considered statistically significant.

Ethical considerations

Written informed consent was obtained from all participants before data collection. Participation was voluntary, and respondents were informed of their right to withdraw at any stage without any consequences. Privacy and confidentiality were maintained throughout the study process. The study did not involve any invasive procedures, and no financial incentives were provided.

## Results

The present study was conducted within the peripheral catchment area of Meerut. A total of 208 participants were included in the study (52 families from each village), which formed the study population. Table 1** **depicts the socio-demographic profile of the 208 study participants.

**Table 1:** Distribution of the study population according to socio-demographic characteristics Data are presented as frequency [n (%)]. Descriptive statistical analysis was performed using frequencies and percentages. No inferential statistical test was applied for this table. OBC: Other Backward Class; SC: Scheduled Caste; HCW: healthcare worker. Socioeconomic status was assessed using the modified B.G. Prasad socioeconomic classification.

Socio-demographic characteristics	Frequency [n (%)]
Age (years)	
≤30	12 (5.8)
31–40	43 (20.7)
41–50	51 (24.5)
51–60	50 (24.0)
61–70	41 (19.7)
71–80	9 (4.3)
≥81	2 (1.0)
Gender	
Female	85 (40.9)
Male	123 (59.1)
Religion	
Hindu	189 (91.0)
Muslim	19 (9.0)
Caste	
General	11 (5.3)
OBC	86 (41.3)
SC	111 (53.4)
Marital status	
Married	174 (83.7)
Widow	34 (16.3)
Education	
Illiterate	51 (24.5)
Primary school	20 (9.6)
Middle school	56 (26.9)
High school	47 (22.6)
Intermediate	20 (9.6)
Graduate	14 (6.7)
Occupation	
Business	13 (6.3)
Daily labour	64 (30.8)
Farmer	26 (12.5)
HCW	9 (4.3)
Housewife	47 (22.6)
Service	13 (6.3)
Unemployed	18 (8.7)
Others	18 (8.7)
Socioeconomic status according to modified B.G. Prasad classification	
Lower middle	1 (0.5)
Middle	10 (4.8)
Upper	113 (54.3)
Upper middle	84 (40.4)

The age distribution shows that the majority of study participants belonged to the middle and older age groups, with the highest proportion in the 41-50 years age group (24.5%), followed closely by 51-60 years (24.0%) and 31-40 years (20.7%). Participants aged 61-70 years constituted 19.7% of the study population. Very young (≤30 years) and very elderly (≥81 years) individuals formed a small proportion, accounting for 5.8% and 1.0% respectively. With regard to gender distribution, males constituted a higher proportion of the study population (59.1%) compared to females (40.9%), indicating a male predominance. In terms of religion, the majority of study participants were Hindus (91%), while Muslims constituted 9% of the study population. Caste-wise distribution revealed that more than half (53.4%) of the study participants belonged to the Scheduled Castes (SC), followed by Other Backward Classes (OBC) (41.3%), while only a small proportion belonged to the General category (5.3%).

Regarding marital status, the majority of study participants were married (83.7%), whereas 16.3% were widows. Educational status showed that a considerable proportion of study participants had low levels of education, with 24.5% being illiterate and 26.9% educated up to middle school. Those with higher education were comparatively fewer, with graduates accounting for only 6.7% of the study population. Occupational distribution indicated that daily labourers formed the largest group (30.8%), followed by housewives (22.6%) and farmers (12.5%). Smaller proportions were engaged in business and service (6.3% each), healthcare work (4.3%), while unemployed and other occupations constituted 8.7% each. Socio-economic status of the family, as per the B.G. Prasad classification, revealed that over half (54.3%) of respondents belonged to the upper socio-economic class, followed by the upper-middle class (40.4%). Only a small proportion of study participants belonged to the middle (4.8%) and lower-middle classes (0.5%). Overall, the study population largely comprised middle-aged individuals, predominantly male, mostly married, with low to moderate educational attainment, engaged mainly in unskilled or semi-skilled occupations, and belonging predominantly to upper and upper-middle socio-economic strata. Table 2illustrates the level of awareness and knowledge of the study population regarding AB-PMJAY.

**Table 2:** Distribution of the study population according to their awareness about AB-PMJAY Data are presented as frequency [n (%)]. Descriptive statistical analysis was performed using frequencies and percentages. No inferential statistical test was applied for this table. AB-PMJAY: Ayushman Bharat Pradhan Mantri Jan Arogya Yojana.

Awareness variables	Frequency [n (%)]
Do you know about/have you heard about AB-PMJAY?	
Yes	201 (96.6)
No	7 (3.4)
Do you know about the benefits of the card?	
Yes	200 (96.2)
No	8 (3.8)
Are you aware of the hospitals empanelled under AB-PMJAY?	
Yes	63 (30.3)
No	145 (69.7)
Are you aware about the availability of services at government and private hospitals?	
Yes	197 (94.7)
No	11 (5.3)
Do you know how to raise a complaint or seek help regarding AB-PMJAY services?	
Yes	55 (26.4)
No	153 (73.6)

An overwhelmingly high proportion of study participants (96.6%) reported that they had heard about or were aware of AB-PMJAY, while only 3.4% were not aware of the scheme. A majority of the population (96.2%) knew about the benefits provided by the Ayushman card. Awareness about hospitals empanelled under AB-PMJAY was relatively low, with only 30.3% of study participants reporting that they were aware of the empanelled hospitals, while a majority (69.7%) lacked this knowledge. In contrast, a very high proportion of study participants (94.7%) were aware that services under AB-PMJAY are available at both government and private hospitals. Awareness about grievance redressal mechanisms was poor. Only 26.4% of participants knew how to raise a complaint or seek help regarding AB-PMJAY services. Table 3 describes the utilization of services under AB-PMJAY among the study population.

**Table 3:** Distribution of participants according to utilization of AB-PMJAY services Data are presented as frequency [n (%)]. Descriptive statistical analysis was performed using frequencies and percentages. No inferential statistical test was applied for this table. AB-PMJAY: Ayushman Bharat–Pradhan Mantri Jan Arogya Yojana.

Utilization variables	Frequency [n (%)]
Are you enrolled under AB-PMJAY?	
Yes	208 (100.0)
Duration since enrolment under AB-PMJAY	
Within 1 year	38 (18.3)
1–3 years	79 (38.0)
More than 3 years	91 (43.8)
Documents used for enrolment	
Aadhaar card only	81 (38.9)
Ration card only	2 (1.0)
Both Aadhaar and the ration card	125 (60.1)
Amount claimed under AB-PMJAY	
≤1 lakh	154 (74.0)
1–3 lakhs	50 (24.0)
3–5 lakhs	4 (1.9)

Regarding the duration since enrolment, the largest proportion of study participants had been enrolled for more than three years (43.8%). This was followed by those enrolled for one to three years (38.0%). A smaller proportion of study participants (18.3%) had been enrolled within the last year. The majority of study participants (60.1%) reported using both Aadhaar and a ration card; Aadhaar alone was used by 38.9% of participants, while only 1.0% used a ration card for enrolment. In terms of financial coverage, most of the study participants (74.0%) reported that their family received coverage of up to Rs. 1 lakh under AB-PMJAY. Nearly one-quarter (24.0%) reported coverage between Rs. 1-3 lakhs, while only a very small proportion (1.9%) availed coverage in the higher range of Rs. 3-5 lakhs. Table 4 presents the distribution of OOP expense incurred by the study population during utilization of services under AB-PMJAY.

**Table 4:** Distribution of participants according to out-of-pocket expenditure incurred during AB-PMJAY utilization Data are presented as frequency [n (%)]. Descriptive statistical analysis was performed using frequencies and percentages. No inferential statistical test was applied for this table. OOPE: out-of-pocket expenditure; INR: Indian Rupees; AB-PMJAY: Ayushman Bharat Pradhan Mantri Jan Arogya Yojana.

OOPE variables	Frequency [n (%)]
Any OOPE incurred?	
Yes	208 (100.0)
Amount spent (INR)	
Rs 0–9,999	164 (78.8)
Rs 10,000–19,999	20 (9.6)
Rs 20,000–29,999	12 (5.8)
Rs 30,000–39,999	8 (3.8)
More than Rs 40,000	4 (1.9)

The majority of participants (78.8%) reported relatively low OOPE, spending less than Rs. 10,000 during their hospitalization. However, a proportion of participants still incurred higher expenditures, with 9.6% spending between Rs. 10,000-19,999 and 5.8% spending Rs. 20,000-29,999. A smaller but noteworthy fraction of participants experienced considerable financial burden, with 3.8% incurring expenses between Rs. 30,000-39,999 and 1.9% spending more than Rs. 40,000 out of pocket.

Table 5 depicts the level of satisfaction among beneficiaries with various aspects of services received under AB-PMJAY.

**Table 5:** Distribution of the study population according to their level of satisfaction regarding services of AB-PMJAY Data are presented as frequency [n (%)]. Descriptive statistical analysis was performed using frequencies and percentages. No inferential statistical test was applied for this table. AB-PMJAY: Ayushman Bharat–Pradhan Mantri Jan Arogya Yojana.

Satisfaction variables	Frequency [n (%)]
Satisfaction with hospital services/quality of treatment	
Dissatisfied	3 (1.4)
Neither satisfied nor dissatisfied	3 (1.4)
Satisfied	202 (97.2)
Satisfaction with behaviour of healthcare staff	
Dissatisfied	1 (0.5)
Neither satisfied nor dissatisfied	1 (0.5)
Satisfied	206 (99.0)
Satisfaction with file management system	
Dissatisfied	36 (17.3)
Neither satisfied nor dissatisfied	135 (64.9)
Satisfied	37 (17.8)
Satisfaction with quality of food provided	
Dissatisfied	19 (9.2)
Neither satisfied nor dissatisfied	159 (76.4)
Satisfied	30 (14.4)

With respect to overall hospital services/quality of service, and quality of treatment, a majority of study participants (97.2%) reported being satisfied, while only a very small proportion (1.4% each) expressed dissatisfaction or neutrality. Satisfaction with the behaviour of healthcare personnel was particularly high. Nearly all study participants (99%) were satisfied with the behaviour. In contrast, satisfaction related to administrative and supportive services was comparatively lower. Regarding the file management system, only 17.8% of study participants reported satisfaction, while a large majority (64.9%) were neither satisfied nor dissatisfied, and 17.3% expressed dissatisfaction. Similarly, satisfaction with the quality of food provided during hospitalization was low, with only 14.4% satisfied, while most participants (76.4%) expressed neutrality, and 9.2% dissatisfaction.

Table 6 shows the association between socio-demographic variables and awareness among beneficiaries regarding AB-PMJAY.

**Table 6:** Associations between beneficiary socio-demographic characteristics and awareness Data are presented as frequency [n (%)]. Inferential statistical analysis was performed using the Chi-square (χ²) test. A p-value of <0.05 was considered statistically significant. AB-PMJAY: Ayushman Bharat–Pradhan Mantri Jan Arogya Yojana; OBC: Other Backward Class; SC: Scheduled Caste.

Socio-demographic characteristics	Awareness (n=208)					χ^2^ (p-value)
	Yes	No		Total		
Religion	Are you aware of the hospitals empanelled under AB-PMJAY?					
Hindu	61 (32.3%)		128 (67.7%)		189(100%)	3.868 (0.049)
Muslim	02 (10.5%)		17 (89.5%)		19(100%)	
Likelihood ratio	4.607 (0.032)					
Bonferroni continuity method	2.906 (0.088)					
	Do you know how to raise a complaint or seek help regarding AB-PMJAY services?					
Hindu	54 (28.6%)		135 (71.4%)		189(100%)	4.822 (0.028)
Muslim	01 (5.3%)		18 (94.7%)		19(100%)	
Likelihood ratio	6.314 (0.012)					
Bonferroni continuity method	3.698 (0.054)					
Caste	Are you aware of the hospitals empanelled under AB-PMJAY?					
General	06 (54.5%)		05 (45.5%)		11 (100%)	13.240 (0.001)
OBC	35 (40.7%)		51 (59.3%)		86 (100%)	
SC	22 (19.8%)		89 (80.2%)		111 (100%)	
Likelihood ratio	13.210 (0.001)					
Education	Are you aware of the hospitals empanelled under AB-PMJAY?					
Illiterate	19 (37.3%)		32 (62.7%)		51 (100%)	13.496 (0.019)
Primary school	03 (15%)		17 (85%)		20 (100%)	
Middle school	12 (21.4%)		44 (78.6%)		56 (100%)	
High school	13 (27.7%)		34 (72.3%)		47 (100%)	
Intermediate	07 (35%)		13 (65%)		20 (100%)	
Graduate	09 (64.3%)		05 (35.7%)		14 (100%)	
Likelihood ratio	13.097 (0.022)					
	Do you know how to raise a complaint or seek help regarding AB-PMJAY services?					
Illiterate	07 (13.7%)		44 (86.3%)		51 (100%)	20.804 (0.001)
Primary school	03 (15%)		17 (85%)		20 (100%)	
Middle school	11 (19.6%)		45 (80.4%)		56 (100%)	
High school	16 (34%)		31 (66%)		47 (100%)	
Intermediate	10 (50%)		10 (50%)		20 (100%)	
Graduate	08 (57.1%)		06 (42.9%)		14 (100%)	
Likelihood ratio	19.975 (0.001)					
Socioeconomic status	Are you aware about the availability of the services at government and private hospitals?					
Upper	110 (97.3%)		03 (2.7%)		113 (100%)	31.712 (<0.001)
Upper middle	80 (95.2%)		04 (4.8%)		84 (100%)	
Middle	07 (70%)		03 (30%)		10 (100%)	
Lower middle	00 (0.0%)		01 (100%)		01 (100%)	
Likelihood ratio	14.008 (0.003)					
	Do you know how to raise a complaint or seek help regarding AB-PMJAY services?					
Upper	41 (36.3%)		72 (63.7%)		113 (100%)	12.581 (0.006)
Upper middle	12 (14.3%)		72 (85.7%)		84 (100%)	
Middle	02 (20%)		08 (80%)		10 (100%)	
Lower middle	00 (0.0%)		01 (100%)		01 (100%)	
Likelihood ratio	13.351 (0.004)					

The values are presented as n (%). Associations were primarily assessed using Pearson’s chi-square test, while the likelihood ratio chi-square was additionally considered. Bonferroni-adjusted values were reported only for specific post hoc 2×2 comparisons generated by the statistical software. The corresponding test statistics and p-values are presented in the table. Awareness of empanelled hospitals was higher among Hindu participants than Muslim participants (32.3% vs 10.5%). This association was statistically significant on Pearson’s chi-square analysis (χ²=3.868, p=0.049), and the likelihood ratio test also suggested significance (LR=4.607, p=0.032); however, the Bonferroni-adjusted value was 0.088, indicating that the association did not retain statistical significance after correction for multiple testing. A similar pattern was seen for awareness of grievance redressal mechanisms, which was reported by 28.6% of Hindu participants and 5.3% of Muslim participants. The association was significant on unadjusted analysis (χ²=4.822, p=0.028; LR=6.314, p=0.012), but the Bonferroni-adjusted value was 0.054, showing that this finding was weakened after correction for multiple comparisons. Caste showed a significant association with awareness of empanelled hospitals (χ²=13.240, p=0.001; LR=13.210, p=0.001). Awareness was highest among participants in the General category (54.5%), followed by Other Backward Classes (40.7%), and lowest among Scheduled Caste participants (19.8%). Education was also associated with awareness of empanelled hospitals (χ²=13.496, p=0.019; LR=13.097, p=0.022). Awareness ranged from 15.0% among participants educated up to primary school to 64.3% among graduates, suggesting better functional awareness of empanelled facilities among more educated respondents. Education showed an even stronger association with awareness of grievance redressal mechanisms (χ²=20.804, p=0.001; LR=19.975, p=0.001). Knowledge of grievance mechanisms increased from 13.7% among illiterate participants to 57.1% among graduates, indicating a marked educational gradient in this domain of awareness. Socioeconomic status was significantly associated with awareness of the availability of services at government and private hospitals (χ²=31.712, p=0.001; LR=14.008, p=0.003). Awareness was highest in the upper (97.3%) and upper-middle (95.2%) groups, but substantially lower in the middle group (70.0%) and absent in the single lower-middle participant. Socioeconomic status was also associated with awareness of grievance redressal mechanisms (χ²=12.581, p=0.006; LR=13.351, p=0.004). Awareness was highest in the upper group (36.3%) and lower in the upper-middle (14.3%) and middle (20.0%) groups, while the only lower-middle participant reported no awareness of the grievance mechanism. Overall, Table 6 indicates that awareness was unevenly distributed across socio-demographic groups, with the clearest differences observed for caste in relation to awareness of empanelled hospitals, for education in relation to grievance redressal awareness, and for socioeconomic status in relation to awareness of service availability. Table 7 shows the association between awareness among beneficiaries and utilization and total OOPE occurred during the utilization of services under AB-PMJAY.

**Table 7:** The association between awareness among beneficiaries, utilization of AB-PMJAY and total OOPE occurred during the utilization of services under AB-PMJAY Data are presented as frequency [n (%)]. Inferential statistical analysis was performed using the Chi-square (χ²) test. A p-value of <0.05 was considered statistically significant. AB-PMJAY: Ayushman Bharat–Pradhan Mantri Jan Arogya Yojana; INR: Indian Rupees.

Utilization	Awareness (n=208)			c^2^ (p-value)
	Yes	No	Total	
	Do you know about benefits of card?			
< 1 Lakh	150 (97.4%)	04 (2.6%)	154 (100%)	6.117 (0.047)
1-3 Lakhs	47 (94%)	03 (6.0%)	50 (100%)	
3-5 Lakhs	03 (75%)	01 (25%)	04 (100%)	
Likelihood ratio	3.522 (0.172)			
Total OOPE (INR)	Do you know about AB-PMJAY?			
Rs 0-9,999	161 (98.2%)	03 (1.8%)	164 (100%)	10.074 (0.039)
Rs 10,000–19,999	19 (95%)	01 (5.0%)	20 (100%)	
Rs 20,000–29,999	11 (91.7%)	01 (8.3%)	12 (100%)	
Rs 30,000–39,999	07 (87.5%)	01 (12.5%)	08 (100%)	
More than Rs 40,000	03 (75%)	01 (25%)	04 (100%)	
Likelihood ratio	5.941 (0.204)			
	Are you aware of the hospitals empanelled under AB-PMJAY?			
Rs 0-9,999	46 (28%)	118 (72%)	164 (100%)	9.846 (0.043)
Rs 10,000–19,999	04 (20%)	16 (80%)	20 (100%)	
Rs 20,000–29,999	05 (41.7%)	07 (58.3%)	12 (100%)	
Rs 30,000–39,999	05 (62.5%)	03 (37.5%)	08 (100%)	
More than Rs 40,000	03 (75%)	01 (25%)	04 (100%)	
Likelihood ratio	9.088 (0.059)			

Awareness of card benefits was highest among beneficiaries with coverage below 1 lakh (97.4%) and lowest among those with 3-5 lakhs coverage (75.0%), with a statistically significant association (p=0.047). However, the likelihood ratio test was not statistically significant (LR=3.522, p=0.172), indicating that this association was not consistently supported across statistical approaches. Awareness of AB-PMJAY also declined as OOPE increased. Beneficiaries spending less than Rs 10,000 showed the highest awareness (98.2%), whereas those with expenditure above Rs. 40,000 showed the lowest awareness (75.0%), and the association was statistically significant (p=0.039). However, the likelihood ratio statistic was not significant (LR=5.941, p=0.204). Awareness of empanelled hospitals showed a significant association (p=0.043) across various OOPE categories. Awareness was highest among respondents with OOPE above Rs 40,000 (75.0%) and lowest among those spending less than Rs. 10,000 (28.0). The likelihood ratio statistic for this association was borderline rather than clearly significant (LR=9.088, p=0.059). Table 8shows the association between the total OOPE occurring during the utilization of services under AB-PMJAY and the utilization of AB-PMJAY.

**Table 8:** The association between total amount of OOPE occurred during the utilization of services under AB-PMJAY and utilization of AB-PMJAY

Total OOPE (INR)	Utilization (n=208)				c^2^ (p-value)
	How much amount coverage does your family get under AB-PMJAY?				
	< 1 lakh	1-3 Lakhs	3-5 Lakhs	Total	
Rs 0-9,999	137 (83.5%)	27 (16.5%)	00 (0.0%)	164 (100%)	65.014 (<0.001)
Rs 10,000–19,999	10 (50%)	10 (50%)	00 (0.0%)	20 (100%)	
Rs 20,000–29,999	04 (33.3%)	06 (50%)	02 (16.7%)	12 (100%)	
Rs 30,000–39,999	01 (12.5%)	06 (75%)	01 (12.5%)	08 (100%)	
More than Rs 40,000	02 (50%)	01 (25%)	01 (25%)	04 (100%)	
Likelihood ratio	47.948 (<0.001)				

Among beneficiaries with OOPE below Rs. 10,000, most had coverage of less than 1 lakh (83.5%), while 16.5% had coverage of 1-3 lakhs and none had coverage of 3-5 lakhs. As OOPE increased, the proportion of beneficiaries with coverage below 1 lakh decreased, while the share in higher coverage categories became more visible. In the Rs. 10,000-19,999 OOPE group, coverage was evenly split between less than 1 lakh and 1-3 lakhs (50% each). In the Rs. 20,000-29,999 group, 33.3% had coverage below 1 lakh, 50% had 1-3 lakhs, and 16.7% had 3-5 lakhs. A similar mixed pattern was seen in the Rs. 30,000-39,999 and >Rs. 40,000 groups, though the numbers were small. This association was statistically significant on both Pearson’s chi-square analysis (χ²=65.014, p=0.001) and likelihood ratio testing (LR=47.948, p=0.001), indicating a clear relationship between expenditure level and coverage utilization in this study population.

Multivariable logistic regression analysis

To account for potential confounding between interrelated sociodemographic variables, multivariable binary logistic regression analysis was performed for selected awareness-related outcomes under AB-PMJAY, and the adjusted model parameters are presented in Table 9. These analyses were undertaken to evaluate whether associations observed on bivariate analysis persisted after simultaneous adjustment for relevant covariates. Because of sparse observations in certain subgroups, some estimates demonstrated wide confidence intervals and should therefore be interpreted cautiously. 

**Table 9:** Multivariable logistic regression model parameters predicting the target clinical outcome across distinct factor groups B: regression coefficient (beta coefficient); S.E.: standard error; Wald: Wald Statistic (the Wald test is used to evaluate the statistical significance of an individual coefficient); OR: odds ratio; CI: confidence interval.

Predictor Variable	B	S.E.	Wald	p-value	OR (95% CI)
MODEL 1: Status of awareness (Do you know about empanelled providers?) on socio-economic factor Sociodemographic Factors (Religion/caste/education)					
Muslim vs. Reference (Hindu)	-2.578	0.929	7.696	0.006	0.076 (0.012, 0.469)
Caste	—	—	15.146	0.001	—
Baseline Reference Category (Scheduled caste)	—	—	—	—	1.000 (Reference)
General vs. Reference	2.640	0.939	7.901	0.005	14.013 (2.224, 88.297)
Other Backward Class vs. Reference	1.218	0.362	11.352	0.001	3.381 (1.665, 6.868)
Education	—	—	5.878	0.318	—
Baseline Reference Category (Illiterate)	—	—	—	—	1.000 (Reference)
Primary school vs. Reference	-1.073	0.714	2.259	0.133	0.342 (0.084, 1.386)
Middle school vs. Reference	-0.852	0.467	3.334	0.068	0.426 (0.171, 1.065)
High school vs. Reference	-0.744	0.471	2.497	0.114	0.475 (0.189, 1.196)
Intermediate vs. Reference	-0.504	0.590	0.730	0.393	0.604 (0.19, 1.92)
Graduate vs. Reference	0.068	0.697	0.009	0.923	1.070 (0.273, 4.191)
Constant	-0.844	0.343	6.059	0.014	0.430
MODEL 2: Status of awareness (Do you know how to raise a complaint or seek help regarding AB-PMJAY services?) on socio-economic factor (socio-economic status/ religion/ education)					
SES of Family (BG Prasad)	—	—	5.062	0.167	—
Baseline Reference Category (Upper)	—	—	—	—	1.000 (Reference)
Upper middle vs. Reference	-0.875	0.396	4.887	0.027	0.417 (0.192, 0.906)
Middle vs. Reference	-0.617	0.848	0.529	0.467	0.540 (0.102, 2.845)
Lower middle vs. Reference	-18.420	40192.97	0.000	1.000	0.000 (0.000, — )
Muslims vs. Reference (Hindu)	-1.637	1.061	2.380	0.123	0.195 (0.024, 1.557)
Education	—	—	9.462	0.092	—
Baseline Reference Category (Illiterate)	—	—	—	—	1.000 (Reference)
Primary school vs. Reference	0.054	0.766	0.005	0.944	1.055 (0.235, 4.735)
Middle school vs. Reference	0.277	0.542	0.260	0.610	1.319 (0.456, 3.814)
High school vs. Reference	0.879	0.530	2.749	0.097	2.407 (0.852, 6.802)
Intermediate vs. Reference	1.315	0.634	4.302	0.038	3.723 (1.075, 12.896)
Graduate vs. Reference	1.615	0.703	5.283	0.022	5.027 (1.269, 19.918)
Constant	-1.200	0.462	6.739	0.009	0.301
MODEL 3: Status of awareness (Do you aware about the availability of the services at government and private hospitals? on socio-economic factor (socio-economic status)					
SES of Family (BG Prasad)	—	—	9.915	0.019	—
Baseline Reference Category (Upper)	—	—	—	—	1.000 (Reference)
Upper middle vs. Reference	-0.606	0.778	0.607	0.436	0.545 (0.119, 2.505)
Middle vs. Reference	-2.755	0.905	9.269	0.002	0.064 (0.011, 0.375)
Lower middle vs. Reference	-24.805	40192.97	0.000	1.000	0.000 (0.000, — )
Constant	3.602	0.585	37.887	<0.001	36.667
MODEL 4: Status of awareness (Do you know the benefits of the card) on the amount covered during utilization					
Amount covered	—	—	4.392	0.111	—
Baseline Reference Category (< 1 Lakh)	—	—	—	—	1.000 (Reference)
1-3 Lakhs vs. Reference	-0.873	0.782	1.246	0.264	0.418 (0.090, 1.934)
3-5 Lakhs vs. Reference	-2.526	1.261	4.012	0.045	0.080 (0.007, 0.947)
Constant	3.624	0.507	51.179	<0.001	37.500
MODEL 5: Status of awareness (Do you know about the AB-PMJAY) on Total OOPE incurred					
Total Amount (Binned)	—	—	6.863	0.143	—
Baseline Reference Category (Rs 0-9,999)	—	—	—	—	1.000 (Reference)
Rs 10,000–19,999 vs. Reference	-1.038	1.180	0.774	0.379	0.354 (0.035, 3.576)
Rs 20,000–29,999 vs. Reference	-1.585	1.196	1.756	0.185	0.205 (0.020, 2.137)
Rs 30,000–39,999 vs. Reference	-2.037	1.218	2.799	0.094	0.130 (0.012, 1.418)
More than Rs 40,000 vs. Reference	-2.884	1.293	4.973	0.026	0.056 (0.004, 0.705)
Constant	3.983	0.583	46.717	<0.001	53.667
MODEL 6: Status of awareness (Do you know about empanelled providers?) on Total OOPE incurred					
Total Amount (Binned)	—	—	8.277	0.082	—
Baseline Reference Category (Rs 0-9,999)	—	—	—	—	1.000 (Reference)
Rs 10,000–19,999 vs. Reference	-0.444	0.585	0.576	0.448	0.641 (0.204, 2.020)
Rs 20,000–29,999 vs. Reference	0.606	0.611	0.983	0.321	1.832 (0.553, 6.066)
Rs 30,000–39,999 vs. Reference	1.453	0.751	3.746	0.053	4.275 (0.982, 18.619)
More than Rs 40,000 vs. Reference	2.041	1.168	3.054	0.081	7.696 (0.780, 75.893)
Constant	-0.942	0.174	29.372	<0.001	0.390

Model 1 evaluated factors associated with awareness of empanelled providers using religion, caste, and education as covariates. After adjustment, Muslim respondents demonstrated significantly lower odds of awareness compared with Hindu respondents (OR=0.076, 95% CI: 0.012-0.469; p=0.006). Caste also remained significantly associated with awareness overall (p=0.001). Compared with Scheduled Caste respondents, participants belonging to the General category (OR=14.013; p=0.005) and Other Backward Class category (OR=3.381; p=0.001) demonstrated higher odds of awareness. Education was not significantly associated with awareness after adjustment (overall p=0.318).

Model 2 examined awareness regarding grievance redressal mechanisms using socioeconomic status, religion, and education as covariates. Socioeconomic status was not significantly associated with the outcome overall after adjustment (p=0.167), although respondents from the upper middle socioeconomic category showed lower odds of awareness compared with the upper class group (OR=0.417, 95% CI: 0.192-0.906; p=0.027). Religion was not independently associated with grievance-related awareness after adjustment (p=0.123). Although the overall association for education did not reach statistical significance (p=0.092), respondents with intermediate education (OR=3.723; p=0.038) and graduate education (OR=5.027; p=0.022) demonstrated significantly higher odds of awareness compared with illiterate participants.

Model 3 assessed awareness regarding the availability of AB-PMJAY services at government and private hospitals according to socioeconomic status. Socioeconomic status remained significantly associated with this awareness outcome overall (p=0.019). Compared with the upper socioeconomic group, respondents in the middle socioeconomic category demonstrated significantly lower odds of awareness (OR=0.064, 95% CI: 0.011-0.375; p=0.002). Estimates for lower socioeconomic subgroups demonstrated wide confidence intervals because of sparse observations.

Model 4 evaluated the association between the amount covered during utilization and awareness of AB-PMJAY card benefits. Although the overall association was not statistically significant (p=0.111), respondents in the Rs. 3-5 lakh coverage category demonstrated lower odds of awareness compared with those in the < Rs. 1 lakh category (OR=0.080, 95% CI: 0.007-0.947; p=0.045). This finding should be interpreted cautiously because of the wide confidence interval and limited subgroup observations.

Model 5 examined the association between total OOPE and general awareness regarding AB-PMJAY. Total OOPE was not significantly associated with awareness overall (p=0.143). However, respondents reporting expenditure > Rs. 40,000 demonstrated lower odds of awareness compared with those reporting OOPE <Rs. 10,000 (OR=0.056, 95% CI: 0.004-0.705; p=0.026). Given the cross-sectional design and sparse observations in higher expenditure categories, these findings should be interpreted cautiously and not as evidence of causal association.

Model 6 assessed the association between awareness of empanelled providers and OOPE levels. The overall association did not reach statistical significance (p=0.082). Although certain expenditure categories demonstrated higher odds ratios, the estimates were imprecise and accompanied by wide confidence intervals. Therefore, these findings should be considered exploratory and hypothesis-generating rather than confirmatory.

## Discussion

This study provides a comprehensive community-based evaluation of beneficiary experiences related to awareness, utilization, satisfaction, and financial burden under AB-PMJAY among rural beneficiaries who had utilized scheme services. Three key findings emerge: first, awareness of the scheme is high but functionally incomplete; second, utilization is substantial but limited in financial depth; and third, financial protection remains partial despite insurance coverage.

The sociodemographic profile of beneficiaries-predominantly middle-aged, male, and belonging to socially disadvantaged groups-aligns with findings from previous studies [13-18]. The predominance of middle-aged individuals is consistent with studies by Sriee et al. [14] and Harish et al. [15], likely reflecting higher healthcare needs and healthcare utilization in this age group. Male predominance observed in the present study mirrors patterns reported by Harish et al. [15] and Shrisharath et al. [16], suggesting possible gender-based differences in healthcare-seeking behavior and access to institutional care. The high representation of Scheduled Castes and Other Backward Classes is also comparable to earlier studies [13,18], indicating that AB-PMJAY is reaching socially vulnerable sections of the population. Educational attainment remained low, similar to observations from other studies [19,20], underscoring the importance of simplified and community-oriented communication strategies. Occupational patterns dominated by daily wage labourers, homemakers, and farmers were likewise consistent with previous reports [21,22], reflecting the scheme’s reach among economically and socially vulnerable households.

An interesting finding of the present study was the relatively higher socioeconomic status of many beneficiaries as assessed by the modified BG Prasad classification. This observation should be interpreted cautiously, as the modified BG Prasad scale is based on current per-capita monthly income adjusted according to contemporaneous Consumer Price Index revisions, whereas AB-PMJAY eligibility is primarily derived from deprivation and occupational criteria recorded in the SECC 2011 database. The latest available updated BG Prasad classification (version Oct 2023) applicable during the study period was used for socioeconomic categorization; however, future studies should continue incorporating periodic Consumer Price Index (CPI)-linked revisions to maintain accurate socioeconomic stratification. Given the substantial interval since SECC enumeration, some households may have experienced improvement in income or living conditions while continuing to remain eligible under the scheme. In addition, the study area included villages with mixed rural-periurban characteristics and relatively stable income sources, which may partly explain the observed socioeconomic distribution. Therefore, higher contemporary socioeconomic classification by the BG Prasad criteria should not necessarily be interpreted as inconsistent with prior eligibility under AB-PMJAY.

Awareness of AB-PMJAY was notably high (96.6%), exceeding levels reported in earlier studies [13,23], and contrasting with findings from Saikia et al. [17], where awareness was substantially lower. Similarly, awareness of scheme benefits was high in the present study, in contrast to poor knowledge reported by Menon et al. [24] and Prasad et al. [19]. However, this awareness was largely superficial. Critical operational knowledge, particularly regarding empanelled hospitals and grievance redressal mechanisms, remained limited, consistent with findings from Prasad et al. [19] and Parekh et al. [25]. This highlights a persistent gap between nominal awareness and actionable knowledge. While most participants were aware that services are available across public and private hospitals, similar to findings by Thomas et al. [26], the lack of knowledge regarding where and how to access these services limits effective utilization.

Utilization patterns in the present study indicate sustained engagement with the scheme, with a majority of participants enrolled for more than one year, consistent with findings by Girish et al. [13]. Aadhaar-based enrolment was predominant, reinforcing its central role in facilitating access. However, the extent of financial utilization was limited, with most beneficiaries reporting coverage utilization below Rs. 1 lakh. This aligns with findings from Girish et al. [13] and Kolekar et al. [27], suggesting that despite broad coverage ceilings, actual utilization remains concentrated at lower expenditure levels. This may reflect limited awareness of entitlements, provider constraints, or barriers in accessing higher-cost services.

A key finding of this study is the persistence of OOPE among all beneficiaries. Although the majority incurred relatively low expenses (< Rs. 10,000), the universal presence of OOPE indicates incomplete financial protection. These findings are consistent with Netra et al. [18], who demonstrated reduced but persistent financial burden among insured populations. More recent evidence by Garg et al. [28] further supports this observation, reporting substantial OOPE and catastrophic expenditure, particularly in private healthcare settings. Similar observations by Harish et al. [15], Thomas et al. [26], and Prasad et al. [19] indicate that non-covered costs-including medicines, diagnostics, food, and transportation-continue to impose financial strain. The present study suggests that residual financial burden persists despite utilization of AB-PMJAY services, particularly because of direct medical and non-medical expenditures not fully covered under the scheme.

Satisfaction levels with clinical services and healthcare provider behaviour were remarkably high, consistent with findings from Parekh et al. [25], Netra et al. [18], and Kanwal et al. [12]. This suggests that the scheme delivers an acceptable quality of clinical care and fosters positive patient-provider interactions. However, satisfaction with administrative and supportive services was notably lower. File management systems and food quality received poor satisfaction ratings, contrasting sharply with findings from Kanwal et al. [12]. This discrepancy highlights variability in institutional performance and underscores the importance of strengthening non-clinical aspects of care, which significantly influence overall patient experience.

The association analyses suggest the presence of sociodemographic gradients in awareness-related outcomes under AB-PMJAY. Awareness regarding empanelled hospitals and grievance mechanisms demonstrated variation across religion, caste, education, and socioeconomic status categories, broadly consistent with earlier studies [17,19]. However, multivariable logistic regression analysis demonstrated attenuation of several crude associations after adjustment, indicating that these relationships are likely influenced by overlapping social and educational determinants rather than isolated demographic effects alone. Similarly, socioeconomic gradients in awareness indicate that informational access remains uneven, potentially reinforcing existing disparities. To further account for potential confounding between interrelated sociodemographic variables, multivariable logistic regression analysis was additionally performed (Table 9). After adjustment, some associations remained statistically significant, whereas others lost significance, indicating that part of the crude association observed on bivariate analysis may be explained by overlapping effects of religion, caste, education, and socioeconomic status. For example, certain awareness outcomes remained independently associated with religion or caste after adjustment, while other associations attenuated after controlling for education and socioeconomic status. These findings suggest that awareness disparities under AB-PMJAY likely reflect broader gradients of social and educational disadvantage rather than isolated demographic effects alone. However, some subgroup estimates demonstrated wide confidence intervals because of sparse observations and should therefore be interpreted cautiously.

**Figure 2:**
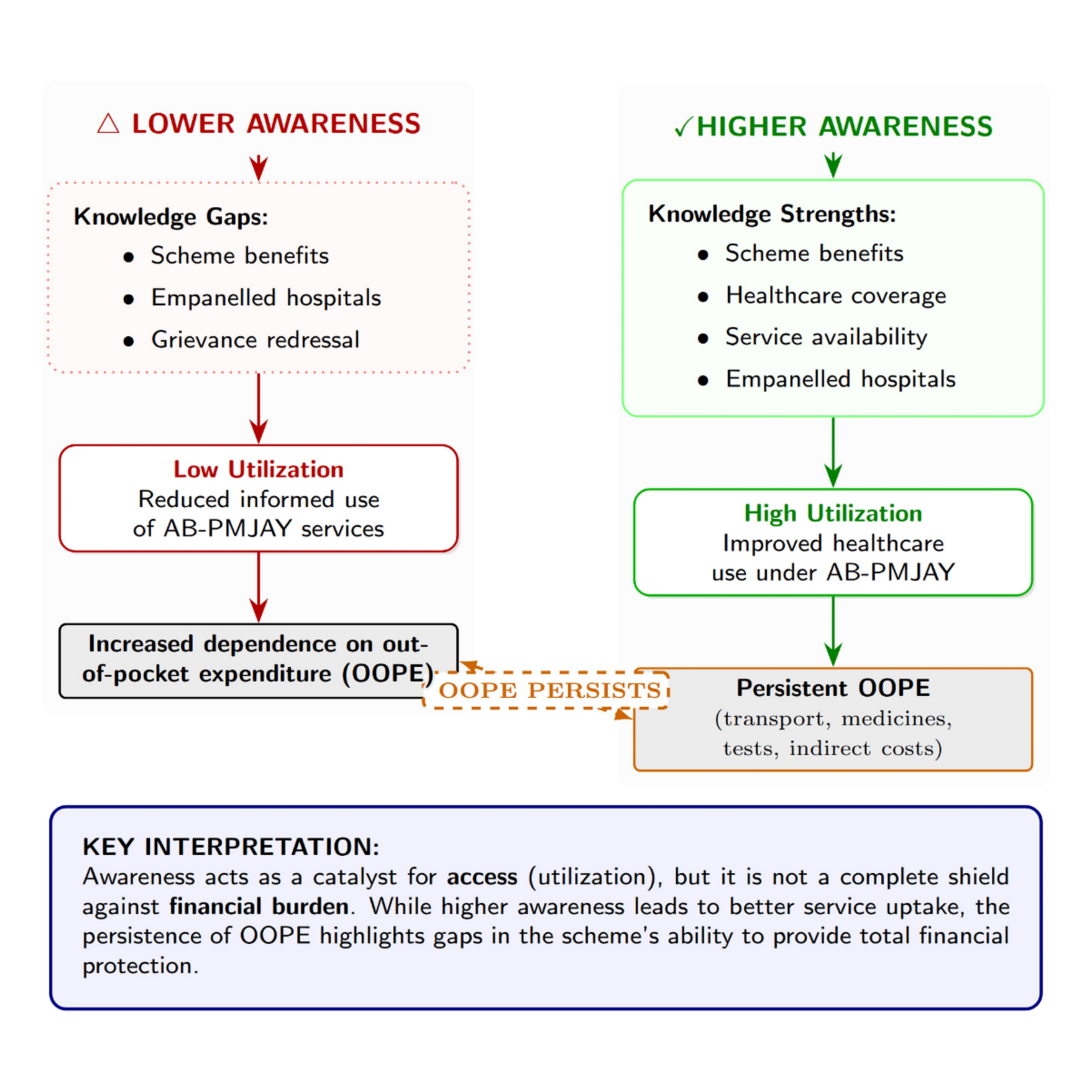
Illustrative schematic showing the association between awareness regarding AB-PMJAY, healthcare utilization, and persistent OOPE among beneficiaries The schematic provides an illustrative representation of the observed relationships between beneficiary awareness regarding AB-PMJAY, healthcare utilization, and residual OOPE among scheme utilizers. The figure highlights that although greater operational awareness was generally associated with improved healthcare-system navigation and utilization, OOPE persisted across awareness levels, suggesting continued residual financial burden despite scheme utilization. The schematic is intended for conceptual visualization of the study findings and should not be interpreted as evidence of a causal relationship. Figure created by the authors using Microsoft PowerPoint 2021. OOPE: out-of-pocket expenditure.

Importantly, awareness of scheme benefits was positively associated with utilization, supporting findings from earlier studies [19,26] that knowledge of entitlements is a key driver of service uptake. However, awareness did not eliminate OOPE. In fact, OOPE persisted across all levels of awareness, consistent with findings from Harish et al. [15], Thomas et al. [26], and Garg et al. [28]. The observed association between awareness and expenditure suggests that while informed beneficiaries may access more services, they may also incur higher incidental costs. However, these associations should be interpreted cautiously because the cross-sectional design does not permit assessment of temporal directionality or causality. The differing patterns observed across awareness domains may reflect varying levels of healthcare-system exposure rather than direct causal effects of awareness on expenditure. Beneficiaries with severe illness, repeated healthcare encounters, referrals, or higher-cost hospitalization episodes may develop greater awareness regarding empanelled hospitals through interaction with the healthcare system, while simultaneously incurring higher incidental and non-covered expenditures. Therefore, the relationship between awareness and OOPE is likely multidimensional and potentially influenced by reverse causality and residual confounding. This highlights a critical limitation: awareness facilitates utilization but does not guarantee financial protection. An illustrative schematic depicting the association between awareness regarding AB-PMJAY, healthcare utilization, and persistent OOPE among beneficiaries has been provided in Figure 2 to facilitate visual interpretation of the observed relationships and key study findings.

Taken together, these findings suggest that AB-PMJAY has made substantial progress in improving awareness, access, and patient satisfaction in rural populations. However, critical gaps remain in functional awareness, administrative efficiency, and financial risk protection. The persistence of OOPE despite universal coverage underscores the need for systemic strengthening, particularly in regulating private sector costs, ensuring availability of medicines and diagnostics, and improving transparency in service delivery. Without addressing these structural gaps, the scheme risks achieving coverage without fully realizing its core objective of financial protection.

Strengths, limitations, and future directions

The present study provides important community-based insights into awareness, utilization patterns, beneficiary satisfaction, and OOPE under AB-PMJAY in a rural Indian setting. One of the major strengths of the study is its field-based design with direct household-level assessment conducted in the community. The use of a standardised semi-structured questionnaire, systematic sampling methodology, and standardized face-to-face interviews conducted directly by the primary investigator helped ensure uniformity in data collection and minimized interviewer-related variability and data collection bias. Additionally, the study evaluated multiple dimensions of AB-PMJAY implementation, including awareness, utilization, healthcare coverage, beneficiary satisfaction, and OOPE, thereby providing a comprehensive assessment of scheme performance at the community level.

However, certain limitations should be acknowledged. Since the study was conducted in only four villages within the rural field practice area of Meerut, the findings may not be fully generalizable to other rural or urban populations. The cross-sectional design limits causal inference between awareness, utilization patterns, satisfaction, and financial burden. Data were collected through participant interviews and were therefore subject to potential recall and response bias. Importantly, the study included only households possessing a valid AB-PMJAY card and having utilized scheme services at least once. Consequently, the findings cannot be generalized to all eligible rural households, particularly those who were unaware of the scheme, unenrolled, or enrolled but never utilized services. Therefore, awareness estimates represent awareness among active beneficiaries rather than community-level awareness prevalence, and the study could not assess barriers related to enrolment or initial non-utilization. Additionally, some subgroup analyses and regression estimates were based on sparse observations and demonstrated wide confidence intervals, which may limit the precision and stability of certain associations. In addition, the questionnaire was designed for pragmatic field-level assessment in a rural community setting and did not employ validated multidimensional psychometric instruments for satisfaction or awareness measurement. Satisfaction was assessed using simplified categorical responses, while awareness was evaluated through operational yes/no items; therefore, the depth and multidimensional complexity of these constructs may not have been fully captured. Certain socioeconomic and healthcare-system factors influencing healthcare-seeking behavior and scheme utilization may also not have been comprehensively assessed.

Future studies should include larger and more diverse populations across multiple rural and urban regions to improve the external validity and generalizability of findings. Multicentric longitudinal studies may provide a better understanding of causal relationships between awareness, healthcare utilization, beneficiary satisfaction, and financial protection under AB-PMJAY. Comparative assessment of public and private empanelled hospitals may further help identify disparities in healthcare delivery, administrative efficiency, and OOPE among beneficiaries. Additionally, qualitative and mixed-method research approaches may provide deeper insights into barriers related to enrolment, healthcare access, grievance redressal mechanisms, and patient experiences, thereby helping guide future policy-level interventions aimed at improving the implementation and effectiveness of AB-PMJAY.

## Conclusions

This study provides community-based insights into beneficiary experiences under AB-PMJAY among rural households that had already utilized scheme services. The findings suggest high beneficiary-level awareness regarding the existence of the scheme and substantial utilization of healthcare services under AB-PMJAY. However, important gaps persisted in functional and operational awareness, particularly regarding empanelled hospitals and grievance redressal mechanisms, which are essential for effective navigation and utilization of scheme benefits. Although most beneficiaries reported relatively low OOPE, residual OOPE persisted despite scheme utilization, indicating continued financial burden related to direct medical and non-medical expenses not fully covered under the scheme. However, in the absence of an uninsured comparison group or pre-enrolment expenditure assessment, the magnitude of financial protection attributable to AB-PMJAY could not be quantified in the present study. Beneficiary satisfaction with clinical care and healthcare provider behaviour was generally high, whereas satisfaction with administrative and supportive services remained comparatively lower, highlighting important operational and service-delivery challenges within the implementation framework. Taken together, these findings suggest that while AB-PMJAY has improved healthcare access and beneficiary experience among active scheme users, strengthening functional awareness, administrative efficiency, grievance support systems, and reducing residual expenditure may further enhance the effectiveness of the scheme in advancing equitable and sustainable financial risk protection.
